# Julia Genz, Paul Gévaudan, editors: Polyphonie in literarischen, medizinischen und pflegewissenschaftlichen Textsorten

**DOI:** 10.3205/zma001523

**Published:** 2022-02-15

**Authors:** Leyla Güzelsoy

**Affiliations:** 1 Klinikum Nürnberg, Klinik für Psychosomatische Medizin und Pyschotherapie, Psychosomatischer Konsiliar- und Liaisondienst & IKP, Nürnberg, Germany; 2 Paracelsus Medizinische Privatuniversität, Nürnberg, Germany

## Bibliographical details

Julia Genz, Paul Gévaudan, editors

**Polyphonie in literarischen, medizinischen und pflegewissenschaftlichen Textsorten**

Vandenhoeck & Ruprecht Verlage

Year of publication: 2021, 243 pages, prize: € 40,00

ISBN: 978-3-8471-0990-7

## Review

For patient-centered medicine, language and communication in healthcare are important factors that are increasingly becoming the focus of research and medical education. The concept of the anthology “Polyphonie in literarischen, medizinischen und pflegewissenschaftlichen Textsorten” (“Linguistic polyphony in literary, medical and nursing text types”) emerged from an interdisciplinary conference on polyphony at the University of Witten/Herdecke in 2018.

The meeting was to provide an opportunity to explore the application of different concepts of linguistic, musical and literary polyphony and their role and integration in different text genres, especially those of health care and medical education. This includes doctor and patient letters and discussions as well as care documentations. Linguistic polyphony is a widespread but also little perceived phenomenon. Polyphony that is not reflected as such can often lead to misunderstandings; and that this is still a field of tension can be seen when reading the volume. The careful use of polyphonic techniques, on the other hand, can lead to a significant improvement in communication, not only in the healthcare sector. 

To start with, the editors provide a short, helpful introduction giving a good overview of the contributions, divided into five sections. The anthology closes with a description of the individual authors.

In the first chapter, “Theoretische Positionen“ (“theoretical positions”), the basics of the theory of polyphony are presented in two linguistic contributions. Current theoretical positions and explanations of Bachtin’s concept of narrative polyphony, published in 1929, which Oswald Ducrot later transferred to argumentative discourses, show the relevance of the topic. Then the basic concepts of linguistic polyphony are analyzed from three points of view and these are transferred into an understandable German terminology.

The second section, “Literarische Polyphonie” (“literary polyphony”), deals with Bachtin’s concept of polyphony in three contributions, both from a music and a literary perspective. It provides a brief historical overview of the five epochs of musical polyphony and their different manifestations. Using literary examples, the concept of the “theory of text interference” and that of the “unreliable narrator” are compared to one another and how they can complement each other. In the last contribution, the linguistic decoding processes of reading are shown, based on novel texts, a content-wise challenging but exciting contribution.

After the first two sections, which focus on the basics of literary studies, chapter three, “Schnittstelle zwischen Literatur und Medizin” (“interface between literature and medicine”), directs the focus to medicine in two very interesting contributions. Julia Genz uses two physicians, Sigmund Freud and Alfred Döblin who were also “literary” and used polyphony in a similar way, but achieved different results. D. Teufel and P. O. Berberat deal with the question to what extent the reflection on polyphony could be integrated into medical studies and show how this could be practiced in a playful way.

The fourth section, “Polyphonie in Arzt – Patienten-Gesprächen” (“polyphony in Doctor-Patient Conversations”), contains four contributions that are specifically based on clinical practice and the use of polyphony there. Beginning with the treatment of oncological patients, juvenile seizure patients and the doctor-patient relationship in general to communication with animals and their owners in veterinary medicine. This last contribution by the author couple Ehlers will be emphasized here, as it gives medical doctors a special insight into communication among and with animals, carried out in an exciting and descriptive way.

Chapter five, “Textsorten und Diskurse im Gesundheitsbereich” (“text types and discourses in the health sector”), with three contributions on the influence of polyphony in patient letters, within the discourse on depression and the nursing documentation, spans another arc into practice and rounds the anthology off very well.

Through careful selection of topics and authors, the editors of this book have succeeded in creating a synthesis between literary studies, linguistics, musicology and health care. The structure of the volume enables to the transfer of profound theoretical knowledge to everyday clinical practice and thus presents the use of polyphony. Through the multi-faceted and clear conveyance of background information, also inexperienced readers are provided with practical aids for everyday clinical practice delivers. Not least in view of the moderate price, this book can be recommended to readers interested in this subject.

## Competing interests

The author declares that she has no competing interests. 

